# Restrained eating in Lebanese adolescents: scale validation and correlates

**DOI:** 10.1186/s12887-021-02728-7

**Published:** 2021-06-01

**Authors:** Tracy Boulos Nakhoul, Anthony Mina, Michel Soufia, Sahar Obeid, Souheil Hallit

**Affiliations:** 1 Faculty of Medicine and Medical Sciences, Holy Spirit University of Kaslik (USEK), Jounieh, Lebanon; 2 INSPECT-LB: National Institute of Public Health, Clinical Epidemiology and Toxicology, Beirut, Lebanon; 3 Faculty of Arts and Sciences, Holy Spirit University of Kaslik (USEK), Jounieh, Lebanon; 4 Research and Psychology Departments, Psychiatric Hospital of the Cross, Jal Eddib, Lebanon

**Keywords:** Restrained eating, Body dissatisfaction, Body mass index, Adolescents, Lebanon, Dutch Restrained Eating Scale, Body dissatisfaction subscale of the Eating Disorder Inventory-Second version (EDI-2), Beirut Distress Scale, Validation

## Abstract

**Background:**

Restrained eating disorder is prevalent worldwide across both ethnic and different cultural groups, and most importantly within the adolescent population. Additionally, comorbidities of restrained eating present a large burden on both physical and mental health of individuals. Moreover, literature is relatively scarce in Arab countries regarding eating disorders, let alone restrained eating, and among adolescent populations; hence, the aim of this study was to (1) validate the Dutch Restrained Eating Scale in a sample of Lebanese adolescents and (2) assess factors correlated with restrained eating (RE), while taking body dissatisfaction as a moderator between body mass index (BMI) and RE.

**Methods:**

This cross-sectional study, conducted between May and June 2020 during the lockdown period imposed by the Lebanese government, included 555 adolescents aged between 15 and 18 years from all Lebanese governorates (mean age of 16.66 ± 1.00 years). The scales used were: Dutch Restrained Eating Scale, body dissatisfaction subscale of the Eating Disorder Inventory-Second version, Rosenberg Self-Esteem Scale, Beirut Distress Scale (for psychological distress), Hamilton Anxiety Rating Scale and Patient Health Questionnaire (for depression).

**Results:**

The Confirmatory factor analysis results were obtained as follows: χ2/df = 159.88/35= 4.57, CFI= 0.96, TLI= 0.95, RMSEA = 0.08 [0.068-0.093]. Female gender (B=0.19), higher BMI (B=0.49), higher physical activity index (B=0.17), following a diet to lose weight (B=0.26), starving oneself to lose weight (B=0.13), more body dissatisfaction (B=1.09), higher stress (B=0.18) were significantly associated with more restrained eating, whereas taking medications to lose weight (B=-0.10) was significantly associated with less restrained eating. The interaction BMI by body dissatisfaction was significantly associated with restrained eating; in the group with low BMI, high body dissatisfaction was significantly associated with more restrained eating. The factor analysis yielded a one-factor solution with Eigen values > 1 (variance explained = 59.65 %; α_Cronbach_ = 0.924). Female gender (B = 0.19), higher BMI (B = 0.49), higher physical activity index (B = 0.17), following a diet to lose weight (B = 0.26), starving oneself to lose weight (B = 0.13), more body dissatisfaction (B = 1.09), and higher stress (B = 0.18) were significantly associated with more RE, whereas taking medications to lose weight (B=-0.10) was significantly associated with less RE. The interaction body mass index (BMI) by body dissatisfaction was significantly associated with RE; in the group with low BMI, higher body dissatisfaction was significantly associated with more RE.

**Conclusions:**

Our study showed that the Dutch Restrained Eating scale is an adapted and validated tool to be used among Lebanese adolescents and revealed factors associated with restrained eating in this population. Since restrained eating has been associated with many clinically-diagnosed eating disorders, the results of this study might serve as a first step towards the development of prevention strategies targeted towards promoting a healthy lifestyle in Lebanese adolescents.

## Background

The term “Eating disorders” represents multiple serious conditions characterized by disordered eating behaviors negatively impacting the physical and mental health of a person, as well as his/her ability to properly function [1]. Eating disorders rank third in terms of chronic diseases [2], are increasing in adolescents [3], are more present in Western compared to Asian countries [4], and are more frequent in females than in males [4].

Among eating disorders, restrained eating (RE) is defined as a behavior “to restrict food intake deliberately in order to prevent weight gain or promote weight loss” [5]. However, some studies indicated that episodes of restrained eating were followed by time intervals of disinhibition towards eating and consequently, weight gain [6, 7]. Moreover, other studies indicated the possibility of stress triggering this alternating restrained/disinhibited eating episode [8, 9]; and hence forming a vulnerable weight cycle [9]. Hormonal changes, along with physical changes and behavioral changes in adolescents, are important factors that might influence the development of restrained eating in these individuals, thus, making it a frequent eating disorder reported in adolescents [10]. Actually, multiple other factors (demographic, social, psychological, etc.) were shown to be associated with restrained eating as well.

### Sociodemographic factors

Females are generally influenced by their body image more than males [11], with adolescent females being less happy about their bodies than adolescent males (20). When comparing boys and girls with the same body mass index (BMI), boys showed more satisfaction of their bodies, while girls were more likely to attempt weight loss maneuvers [11].

### Physical activity

A correlation seems to exist between physical activity and restrained eating. The results of a previous study indicated that girls at high risk of developing an eating disorder, performed more physical activity, with the goal of losing weight [12]. Indeed, more eating restriction was observed in adolescents who practiced more physical activity [12].

### Social factors

Social factors include family, parents and media. A previous study demonstrated that teasing by family and friends, as well as internalized weight stigma, especially that related to weight and body shame and guilt, are correlated with eating disorder [12]. Parents, family and media can lead the individual to restrained eating by influencing him/her to reach the “perfect weight” [13, 14]. An illustrative example is that excessive watching of reality and entertainment shows leads to disordered eating in women [15]. Media perfectionism, and media pressure increase the occurrence of body dissatisfaction that can lead to restrained eating [16]. Even though parents are a source of social support, they can increase teens’ body dissatisfaction by criticizing their appearance, hence, contributing to the development of restrained eating [17].

### Psychological factors

Among those factors emerges body dissatisfaction. Its core “negative feeling about the body” affects restrained eating both directly and indirectly by different pathways [8, 18, 19]. Having thinner body ideals as a tool for better social recognition among peers, is a well-established concept among adolescents, paving way for more body dissatisfaction and increased effort to lose weight among them [20]. Furthermore, adolescents with a high BMI level are more likely to get dissatisfied with their current weight, and become prone to weight decreasing maneuvers, particularly restrained eating [18].

Another psychological factor is depression. There is a positive correlation between depression and restrained eating in women, but not men [18]. In women, the positive effect of depression on restrained eating suggests that focusing on food, eating or dieting may be a broader group of methods for escaping awareness of negative emotions [17]. In men, depression inhibits, rather than facilitates, restrained eating, suggesting that men do not use food to regulate their emotions, and do not rely on diet to escape unwanted emotions [17, 18].

**Fig. 1:**
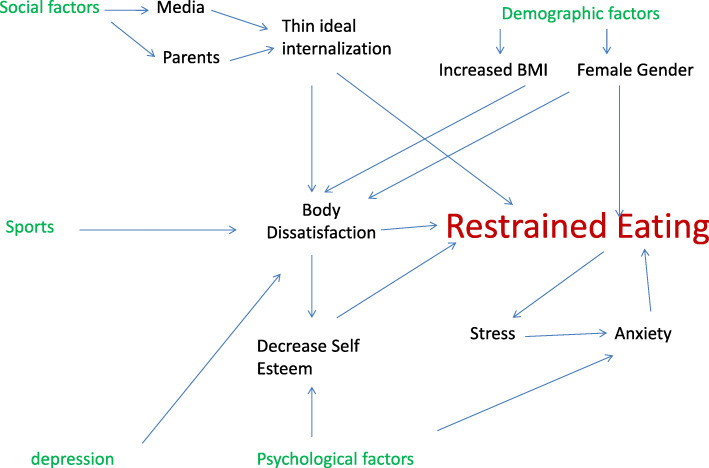
Theoretical framework showing factors associated with restrained eating and their interaction [11, 18, 21, 22]

Thus, based on previous studies [11, 18, 21, 22], body dissatisfaction seems to play a moderating role between multiple factors (body mass index, physical activity, depression, self-esteem, gender) and restrained eating. Based on the before mentioned studies, the following trans-theoretical model of restrained eating was developed (Fig. 1).

Multiple scales are used for the assessment of restrained eating: Dutch Eating Behavior Questionnaire, Eating Inventory (EI), Revised Restraint Scale (RS), and the Current Dieting Questionnaire [23]. The Dutch Eating Behavior Questionnaire (DEBQ), originally developed by Van Strien et al. in 1986, assesses restrained, emotional, and external eating behavior [24]. In addition, subsequent exploratory and confirmatory factor analyses generally have supported the original three-factor structure [25]. The DEBQ has equivalent psychometric properties and factor structure in men and women and across the full range of weight categories. In this study, only the restrained questionnaire will be adopted. The Dutch Restrained Eating Scale (DRES) was validated in different languages in adolescents’ populations, mainly French [26], Maltese [27] and Spanish [28], with the last two studies validating the DRES in female gender exclusively [27, 28]. A study conducted in Lebanon about restrained eating did validate the Arabic version of the Dutch Restrained Eating Scale in adults [29].

Restrained eating disorder is prevalent worldwide across both ethnic and different cultural groups [30] and most importantly within the adolescent population [31]. Additionally, comorbidities of restrained eating present a large burden on both physical and mental health of individuals [32]. Moreover, literature is relatively scarce in Arab countries on eating disorder, let alone restrained eating, and in adolescents populations; hence, the aim of this study was to (1) validate the Arabic version of the Dutch Restrained Eating Scale and (2) assess factors associated with restrained eating among a sample of Lebanese adolescents, while taking body dissatisfaction as a moderator between BMI and RE.

## Methods

### Study design

This cross-sectional study, conducted between May and June 2020 during the lockdown period imposed by the Lebanese government, included 555 adolescents aged between 15 and 18 years old from all Lebanese governorates (Beirut, Mount-Lebanon, South, North, Bekaa). Our sample was chosen using the snowball technique; the research team contacted adolescents in their contact lists from different schools; those students were instructed to forward the link to the questionnaire to their classmates via the WhatsApp application. The first page of the questionnaire included an explanation of the study topic and objective, a statement ensuring the anonymity of respondents and an explanation for the student to get his/her parents’ approval before participation. The student had to select the option stating “I got my parents’ approval and I consent to participation in this study” to be directed to the questionnaire.

The mean age of the participants was 16.66 ± 1.00 years, with 75.7 % females. More details about the students can be found in Table 1. The mean restrained eating score in the total sample was 26.30 ± 9.37 (skewness=0.203; kurtosis= -0.656).

**Table 1:** Sociodemographic and other characteristics of the participants (*N* = 555)

Variable	N (%)
**Gender**	
Male	135 (24.3 %)
Female	420 (75.7 %)
**District**	
Beirut	62 (11.2 %)
Mount Lebanon	332 (59.8 %)
North	92 (16.6 %)
South	29 (5.2 %)
Bekaa	40 (7.2 %)
	**Mean ± SD**
Age (in years)	16.66 ± 1.00
Body Mass Index (kg/m2)	22.38 ± 4.00
House crowding index	0.99 ± 0.52

### Minimal sample size

Since the Dutch Restrained Eating Scale contains 10 items, a sample of 100 adolescents was deemed necessary to conduct a factor analysis (10 participants per 1 scale item according to Comrey and Lee) [33].

### Questionnaire

The first part of the questionnaire contained socio-demographic information about the participants (age, gender, governorate, current weight and height). The household crowding index, reflecting the socioeconomic status of the family [34], is the ratio of the number of persons living in the house over the number of rooms in it (excluding the kitchen and the bathrooms). The physical activity index is the cross result of the intensity, duration, and frequency of daily activity [35].

The second part included the scales used in this study:

#### Dutch Restrained Eating Scale

It is composed of 10 questions [36] rated from never (1 point) to always (5 points). Higher scores reflect more restrained eating (Cronbach’s α in this study = 0.92).

#### Body dissatisfaction subscale of the Eating Disorder Inventory-Second version (EDI-2)

It is composed of nine items, scored from 0 (sometimes/rarely/never) to 3 (always). Higher scores define more body dissatisfaction (Cronbach’s α in this study = 0.63) [37].

#### Rosenberg Self-Esteem Scale

This scale is used to assess self-esteem [38]. It includes ten items measured on 4-point Likert scale ranging from 1 (strongly disagree) to 4 (strongly agree). Higher scores reflected higher self-esteem (Cronbach’s α in this study = 0.74).

#### Beirut Distress Scale (BDS-10)

This scale, developed in Lebanon [39], was used to assess the intensity of distress. It is composed of ten questions. The points range from 0 (never) to 4 (always). A higher score indicates higher perceived distress (Cronbach’s α in this study = 0.82).

#### Hamilton Anxiety Rating Scale (HAM-A)

It is composed of fourteen items rated on a 5-point Likert scale ranging from 0 (not present) to 4 (very severe) [40]. Higher scores mean more anxiety. This scale is validated in the Lebanese population [41] (Cronbach’s α in this study = 0.89).

#### Patient Health Questionnaire (PHQ-9)

This nine-item scale was used to assess depression [42] and is validated in Lebanon [43]. Scores range from 0 (not at all) to 3 (nearly every day). Higher scores indicate higher rates of depression (Cronbach’s α in this study = 0.84).

The last part of the questionnaire included general questions retrieved from a previous study [44] about methods to lose weight, dieting, food, external pressures to go on a diet, abuse and family history of eating disorder (i.e. “do you always hear a comment about your weight?”, “do your relatives comment on your weight?”, “do you feel pressured to go on a diet?”, “have you felt pressured by the media to change your diet?”, “have you followed any diet to lose weight?”). These variables were classified as categorical variables (yes/no type of answer).

### Forward and back translation

One bilingual psychologist whose mother tongue is Arabic, accomplished the forward translation. The backward translation was performed by another psychologist. The original and translated English versions were compared by one healthcare professional (psychiatrist) for discrepancies, which were resolved by consensus [45–51].

### Statistical analysis

The SPSS software version 23 was used to conduct data analysis. A confirmatory factor analysis (CFA) was carried out on the total sample using SPSS AMOS software v24. Several goodness-of-fit indicators were reported: the Relative Chi-square (χ2/df), the Root Mean Square Error of Approximation (RMSEA), the Tucker Lewis Index (TLI) and the comparative fit index (CFI). The value of χ2 divided by the degrees of freedom (χ2/df) has a low sensitivity to sample size and may be used as an index of goodness of fit (cut-off values: < 2–5). Values of RMSEA of 0.08 or less indicate a good-fitting model, while TLI and CFI values greater than 0.90 indicate excellent model fit [53]. Cronbach’s alpha values ensured internal reliability of the scales.

The normality of distribution of the restrained eating score was confirmed via a calculation of the skewness and kurtosis; values for asymmetry and kurtosis between − 2 and + 2 are considered acceptable in order to prove normal distribution [53]. These conditions consolidate the assumptions of normality in samples larger than 300 [54]. The Student t and ANOVA tests were used to compare two and three or more means respectively. Pearson correlation was used to correlate two continuous variables. A multiple stage set of linear regressions was conducted, taking the restrained eating score as the dependent variable and all variables that showed a *p* < 0.05 in the bivariate analysis as independent variables. Sociodemographic characteristics were entered at the first step; as a second step, practices followed by the participants (vomiting/starving/medications to lose weight, etc.) were entered as independent variables; at the third step, anxiety, depression, stress and body dissatisfaction were entered in the model; finally, the interaction BMI by body dissatisfaction was entered as an independent variable. *P* < 0.05 was considered significant.

## Results

### Confirmatory factor analysis

The CFA results confirmed the one-factor structure of the restrained eating scale as follows: χ2/df = 159.88/35= 4.57, CFI= 0.96, TLI= 0.95, RMSEA = 0.08 [0.068-0.093]. The standardized loading factors are summarized in Table 2.

**Table 2:** Standardized coefficients and standard errors of the Dutch Restrained Eating Scale

	Standard errors	Standard errors	*P*
Item 1	0.72	-	-
Item 2	0.64	0.06	<0.001
Item 3	0.75	0.06	<0.001
Item 4	0.51	0.06	<0.001
Item 5	0.65	0.06	<0.001
Item 6	0.77	0.06	<0.001
Item 7	0.85	0.06	<0.001
Item 8	0.85	0.06	<0.001
Item 9	0.80	0.06	<0.001
Item 10	0.77	0.06	<0.001

### Bivariate analysis

Higher anxiety, depression, stress, body dissatisfaction, higher physical activity index and BMI were significantly associated with more restrained eating (Table 3). Female gender, following a diet to lose weight, those who weigh themselves daily those who starve themselves to lose weight, and those who feel pressured by media to lose weight had significantly more restrained eating (Table 4).

**Table 3:** Bivariate analysis of continuous variables associated with the restrained eating score

Variable	Correlation coefficient
Body dissatisfaction	*r* = 0.365; *p* < 0.001
Self-esteem	*r* =-0.038; *p* = 0.376
Anxiety	*r* = 0.187; *p* < 0.001
Depression	*r* = 0.183; *p* < 0.001
Stress	*r* = 0.218; *p* < 0.001
Age	*r* =-0.02; *p* = 0.640
House crowding index	*r* =-0.057; *p* = 0.181
Body Mass Index	*r* = 0.245; *p* < 0.001
Physical activity index	*r* = 0.168; *p* < 0.001

**Table 4:** Bivariate analysis of categorical variables associated with the restrained eating score

Variable	Mean restrained eating score
**Gender**	
Male	22.09 ± 8.71
Female	27.65 ± 9.18
* P*	**0.001**
**Following a diet to lose weight**	
No	23.13 ± 7.98
Yes	31.40 ± 9.20
* P*	**< 0.001**
**Vomiting to lose weight**	
No	25.96 ± 9.06
Yes	28.68 ± 11.15
* P*	0.056
**Taking medications to lose weight**	
No	26.40 ± 9.23
Yes	25.27 ± 10.73
* P*	0.412
**Starving to lose weight**	
No	24.79 ± 8.68
Yes	31.04 ± 9.91
* P*	**< 0.001**
**Weighing yourself daily**	
No	25.40 ± 8.82
Yes	28.86 ± 10.39
* P*	**< 0.001**
**Family history of eating disorders**	
No	25.63 ± 9.39
Yes	28.14 ± 9.11
* P*	**0.005**
**Pressured by media to lose weight**	
No	24.58 ± 9.36
Yes	29.40 ± 8.59
* P*	**< 0.001**

Restrained eating score was calculated from the Dutch Restrained Eating Scale; numbers displayed in the table represent correlation coefficients obtained from the Pearson correlation test.

### Multivariable analysis

**Fig. 2:**
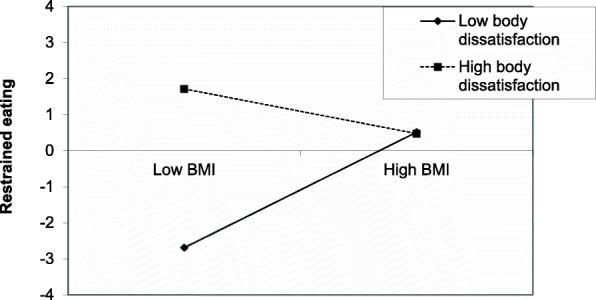
Association between the interaction of body mass index with body dissatisfaction on restrained eating

The results of the hierarchical linear regressions are shown in Table 5. In the final model that included all independent variables, female gender (B = 0.19), higher BMI (B = 0.49), higher physical activity index (B = 0.17), following a diet to lose weight (B = 0.26), starving oneself to lose weight (B = 0.13), more body dissatisfaction (B = 1.09), higher stress (B = 0.18) were significantly associated with more restrained eating, whereas taking medications to lose weight (B=-0.10) was significantly associated with less restrained eating (Table 5, Model 4). The interaction BMI by body dissatisfaction was significantly associated with restrained eating; in the group with low BMI, high body dissatisfaction was significantly associated with more restrained eating (Fig. 2).

**Table 5:** Multivariable analysis: Linear regression taking the restrained eating score as the dependent variable

**Variable**	**Unstandardized Beta**	**Standardized Beta**	***p***	**95 % CI**
**Model 1: sociodemographic variables as independent variables.**				
Gender (females vs. males*)	7.02	0.32	**< 0.001**	4.36–8.68
Body Mass Index	0.69	0.29	**< 0.001**	0.51–0.87
Family history of eating disorders (yes vs. no*)	1.43	0.07	0.083	-0.19-3.05
Physical activity index	0.08	0.23	**< 0.001**	0.05–0.10
Nagelkerke *R*^2^ = 20.2 %				
**Model 2: sociodemographic variables and practices followed as independent variables.**				
Gender (females vs. males*)	5.25	0.24	**< 0.001**	3.66–6.85
Body Mass Index	0.49	0.21	**< 0.001**	0.32–0.67
Family history of eating disorders (yes vs. no*)	0.74	0.04	0.353	0.83 − 2.32
Physical activity index	0.06	0.18	**< 0.001**	0.04–0.08
Following a diet to lose weight (yes vs. no*)	5.06	0.30	**< 0.001**	4.22–7.23
Medication intake to lose weight (yes vs. no*)	-5.06	-0.10	**0.004**	-5.71- -1.05
Starving oneself to lose weight (yes vs. no*)	2.95	0.13	**0.001**	1.06–4.44
Pressured by media to lose weight (yes vs. no*)	0.36	0.04	0.370	-0.80-2.15
Weighing yourself daily (yes vs. no*)	2.04	0.10	0.016	-0.39-3.68
Nagelkerke *R*^2^ = 32.4 %				
**Model 3: sociodemographic variables, practices followed, mental health variables and body dissatisfaction and self-esteem as independent variables.**				
Gender (females vs. males*)	4.82	0.22	**< 0.001**	3.23–6.41
Body Mass Index	0.36	0.16	**< 0.001**	0.18–0.55
Family history of eating disorders (yes vs. no*)	-0.001	0.001	0.999	-1.55-1.55
Physical activity index	0.06	0.17	**< 0.001**	0.03–0.08
Following a diet to lose weight (yes vs. no*)	4.57	0.24	**< 0.001**	3.01–6.13
Medication intake to lose weight (yes vs. no*)	-4.60	-0.14	**< 0.001**	-7.06- -2.14
Starving oneself to lose weight (yes vs. no*)	3.00	0.14	**0.001**	1.26–4.73
Pressured by media to lose weight (yes vs. no*)	-0.49	-0.03	0.532	-2.05-1.06
Body dissatisfaction	1.77	0.08	**0.031**	0.17–3.37
Self-esteem	0.26	0.18	**< 0.001**	0.14–0.39
Anxiety	0.04	0.05	0.324	-0.04-0.12
Depression	-0.15	-0.09	0.124	-0.33-0.04
Stress	0.33	0.20	**< 0.001**	0.16–0.50
Nagelkerke *R*^2^ = 37.4 %				
**Model 4: sociodemographic variables, practices followed, mental health variables and body dissatisfaction, self-esteem and the interaction of body dissatisfaction with BMI as independent variables.**				
Gender (females vs. males*)	4.93	0.23	**< 0.001**	3.36–6.50
Body Mass Index	1.14	0.49	**< 0.001**	0.71-1.57
Family history of eating disorders (yes vs. no*)	-0.01	0.001	0.989	-1.52-1.54
Physical activity index	0.05	0.16	**< 0.001**	0.03–0.08
Following a diet to lose weight (yes vs. no*)	4.50	0.23	**< 0.001**	2.95-6.04
Medication intake to lose weight (yes vs. no*)	-4.73	-0.15	**< 0.001**	-7.16- -2.30
Starving oneself to lose weight (yes vs. no*)	3.04	0.14	**0.001**	1.32-4.76
Pressured by media to lose weight (yes vs. no*)	-0.42	-0.02	0.591	-1.95-1.12
Weighing yourself daily (yes vs no*)	1.59	0.08	**0.049**	0.01-3.18
Body dissatisfaction	1.43	0.98	**< 0.001**	0.83-2.03
Self-esteem	0.21	0.12	**0.002**	0.07-0.34
Anxiety	0.04	0.04	0.340	-0.04-0.12
Depression	-0.13	-0.07	0.179	-0.31-0.06
Stress	0.31	0.19	**< 0.001**	0.15-0.48
Interaction BMI by body dissatisfaction	-0.05	-0.99	**< 0.001**	-0.08- -0.03
Nagelkerke *R*^2^ = 39.1 %

## Discussion

### Scale validation

In our study, the DRES converged over a solution of one factor similarly to the study conducted among Lebanese adults [29] and Maltese female adults [27]. The Cronbach’s alpha in our study is 0.924, reflecting an excellent internal consistency. We observed equivalent results by Van Strien et al. in the original DRES with alpha between 0.8 and 0.95 [29]. Also Cronbach’s alpha was 0.87 in the Maltese version [27] and 0.9 in the Spanish version [28]. However, the RMSEA value obtained in the confirmatory factor analysis is borderline and might not show adequate fit indices. Thus, the Arabic version of the Dutch Restrained Eating Scale is similar to the original version and might be considered a reliable tool for Lebanese adolescents. Future studies are needed for the assessment of additional psychometric properties of the Arabic version.

### Correlates of restrained eating

To our knowledge, this is the first study done in Lebanon assessing factors associated with restrained eating in adolescents. Results showed that female gender, having a higher BMI, practicing more physical activity, following a diet to lose weight, starving oneself, higher body dissatisfaction and stress, were associated with more restrained eating. Taking medications to lose weight was associated with less restrained eating. The interaction BMI by body dissatisfaction turned out to be significantly associated with restrained eating. *Gender*.

This study results showed that female gender was significantly associated with more restrained eating, in line with previous studies [11, 55]. This might be due to the social pressure exerted on females, and the expectation of an ideal body, which leads girls to restrain from eating [11]. Moreover, with older age, a higher rate of body dissatisfaction was observed in females compared to their male counterparts [11]. While girls have more plans to decrease their weight, boys focus more on increasing weight and muscle building [11]. Girls might be more concerned with comparing their bodies to the ideal ones shown in media [11]. They seek the stereotype of beautiful thin women in order to confirm that they meet social expectation of femininity, consequently leading them to more restrained eating [11].

### Body Mass Index

In our study, a significant positive relationship between BMI and restrained eating was established, in line with previous findings [10]. The higher the BMI of an adolescent, the higher the risk of dieting [10]. The mechanism by which BMI leads to restrained eating is not fully explained [10]. When individuals reach puberty, weight gain happens due to hormonal changes; individuals will thrive to become thinner and will go on a diet.

### Body dissatisfaction

Our results showed a positive association between body dissatisfaction and restrained eating, in line with previous studies [8, 18, 19]. This could be explained by the fact that adolescents who have a high BMI are ashamed of their bodies, developing a negative image about themselves, resulting in restrained eating in order to decrease their weight [18].

In addition, our results showed that the interaction BMI by body dissatisfaction was significantly associated with restrained eating; in the group with low BMI, high body dissatisfaction was significantly associated with more restrained eating. To our knowledge, in adults, higher weight suppression (WS), defined as the difference between maximal and current weight, is associated with decreased leptin and loss of control eating [56] and more body dissatisfaction [57]. The social stigma associated with obesity may cause shame, guilt and body dissatisfaction [58]. This is clinically important since body dissatisfaction is an unpleasant result of obesity, which serves as a motivation to follow unhealthy eating behaviors and weight control practices [59].

### Physical activity

Our results showed that higher physical activity was related to more restrained eating, in line with a previous study that showed that exercise was able to favorably modify the short-term appetite control [60]. It is important to note that physical activity does not always imply going to a gym. During the COVID-19 pandemic, it is more frequent that adolescents exercise outdoor, or at home, with online/internet videos. The association between restrained eating and physical activity in determining energy intake after exercise, remains unclear and may be related to disinhibition (loss of restraint) levels [61]. Restrained eaters tend to decrease their energy intake after exercise, which creates a negative energy balance; the opposite is true about unrestrained eaters who actually increase their energy intake after physical activity [62]. More studies are recommended to solve the mystery of this dilemma.

### Following a diet

Our study showed a positive association between following a diet and restrained eating, in line with previous findings [63]. People who follow a diet learn how to do self-control and have previous success in this regard, which makes them successful restrained eaters [64].

### Starving oneself

Our study demonstrated a positive significant association between starving one self and restrained eating. In the general population, starving oneself does not precede restrained eating. In fact, previous findings [65, 66] showed that starving oneself and eating restriction are two behaviors that occur at the same time in order to lose weight. Controversially, another study demonstrated that dieting and restrained eating increase starvation and food cravings [67]. Therefore, our results should be interpreted with caution.

### Stress

In our study, a positive relationship was established between stress and restrained eating. Literature is controversial in this regard; while previous studies showed that overeating can be used as a compensation of stress and negative mood [68, 69], other findings showed that stress can result in undereating [70]. More studies are needed to clarify this association.

### Medication to lose weight

Our study showed a negative association between restrained eating and the use of medication to lose weight. No previous studies regarding this association have been conducted in adolescents. We hypothesize that this negative association could be due to the fact that when adolescents take medications to lose weight, they rely on them and do not restrict their eating because they think that medications alone are more than enough to control their weight [71]. At the same time, not restricted eating does not imply overeating. Those results should also be interpreted with caution.

## Limitations

This study is a cross-sectional study, therefore we cannot conclude causality. It is important to point out that the confirmatory factor analysis results did not show adequate fit indices. Diagnosis was made by means of a questionnaire (less accurate) rather than a clinical interview. Females outnumbered males; furthermore, the majority of adolescents recruited went to school, and were from Mount Lebanon. The recruitment strategy (snowball technique) does not guarantee representativeness and extrapolation of our results to the general population. The questions used in the chosen scales addressed females more than males (questions referring to part of the body from the waist and downwards). Some scales (self-esteem and body dissatisfaction) have not been validated in Lebanon so far. A residual confounding bias is also possible since not all factors associated with restrained eating were considered in this study. Finally, we did not add another scale assessing restrained eating to help estimate the construct validity of the DRES in Lebanon. Future studies taking all these limitations into consideration are needed.

## Conclusions

In conclusion, our study presents preliminary results for the validation of the Dutch restrained eating scale among Lebanese adolescents and revealed factors associated with restrained eating in this group. This study, by validating the DERS in Lebanese adolescents, would help clinicians detect harmful eating practices (restrained eating) in persons within this age group. The results of this study might serve as a first step towards the development of prevention strategies targeted towards promoting a healthy lifestyle in Lebanese adolescents.

## Data Availability

All data generated or analyzed during this study are not publicly available to maintain the privacy of the individuals’ identities. The dataset supporting the conclusions is available upon request to the corresponding author.

## Abbreviations

BMI: Body mass index
EI: Eating Inventory
RRS: Revised Restraint Scale
DEBQ: Dutch Eating Behavior Questionnaire
DERS: Dutch Restrained Eating Scale
RE: Restrained eating
BDS: Beirut Distress Scale
HAM-A: Hamilton Anxiety Rating Scale
PHQ-9: Patient Health Questionnaire
FA: Factor analysis
CFA: Confirmatory factor analysis
KMO: Kaiser–Meyer–Olkin
RMSEA: Root Mean Square Error of Approximation
GFI: Goodness of Fit Index
AGFI: Adjusted Goodness of Fit Index
