# Correction to: Restrained eating in Lebanese adolescents: scale validation and correlates

**DOI:** 10.1186/s12887-022-03211-7

**Published:** 2022-04-28

**Authors:** Tracy Boulos Nakhoul, Anthony Mina, Michel Soufia, Sahar Obeid, Souheil Hallit

**Affiliations:** 1 Faculty of Medicine and Medical Sciences, Holy Spirit University of Kaslik (USEK), Jounieh, Lebanon; 2 INSPECT-LB: National Institute of Public Health, Clinical Epidemiology and Toxicology, Beirut, Lebanon; 3 Faculty of Arts and Sciences, Holy Spirit University of Kaslik (USEK), Jounieh, Lebanon; 4 Research and Psychology Departments, Psychiatric Hospital of the Cross, Jal Eddib, Lebanon

**Correction to: BMC Pediatr 21, 257 (2021)**

**https://doi.org/10.1186/s12887-021-02728-7**

Following publication of the original article [1], the authors flagged that they had based their analysis on the whole sample collected for their study, but that some participants should have been excluded from the sample: *N* = 614 had been given, while it should be *N* = 555.

To correct this, the authors have redone their analysis on the corrected sample of *N* = 555 and the original article has been corrected according to the new analysis; please see the additional file provided in this correction for the details of the corrections that have been made (the yellow highlighting indicates the updates).

The authors thank you for reading this correction and apologize for any inconvenience caused.

## Supplementary Information

**Additional file 1.**
